# A plant endophyte *Staphylococcus hominis* strain MBL_AB63 produces a novel lantibiotic, homicorcin and a position one variant

**DOI:** 10.1038/s41598-021-90613-9

**Published:** 2021-05-27

**Authors:** M. Aftab Uddin, Shammi Akter, Mahbuba Ferdous, Badrul Haidar, Al Amin, A. H. M. Shofiul Islam Molla, Haseena Khan, Mohammad Riazul Islam

**Affiliations:** 1 Molecular Biology Laboratory, Department of Biochemistry and Molecular Biology, Faculty of Biological Sciences, University of Dhaka, Dhaka, 1000 Bangladesh; 2 Department of Genetic Engineering and Biotechnology, Faculty of Biological Sciences, University of Dhaka, Dhaka, 1000 Bangladesh; 3 Plant Biotechnology Division, National Institute of Biotechnology, Ganakbari, Ashuliya, Savar, Dhaka, 1349 Bangladesh; 4 Divisional DNA Screening Laboratory, Sylhet MAG Osmani Medical College Hospital, Sylhet, 3100 Bangladesh; 5 Institute of National Analytical Research and Service, Bangladesh Council of Scientific and Industrial Research (BCSIR), Dhaka, 1205 Bangladesh

**Keywords:** Drug discovery, Microbiology

## Abstract

Here we report a jute endophyte *Staphylococcus hominis* strain MBL_AB63 isolated from jute seeds which showed promising antimicrobial activity against *Staphylococcus aureus* SG511 when screening for antimicrobial substances. The whole genome sequence of this strain, annotated using BAGEL4 and antiSMASH 5.0 to predict the gene clusters for antimicrobial substances identified a novel antimicrobial peptide cluster that belongs to the class I lantibiotic group. The predicted lantibiotic (homicorcin) was found to be 82% similar to a reported peptide epicidin 280 having a difference of seven amino acids at several positions of the core peptide. Two distinct peaks obtained at close retention times from a RP-HPLC purified fraction have comparable antimicrobial activities and LC–MS revealed the molecular mass of these peaks to be 3046.5 and 3043.2 Da. The presence of an oxidoreductase (*homO*) similar to that of epicidin 280- associated *eciO* or epilancin 15X- associated *elxO* in the homicorcin gene cluster is predicted to be responsible for the reduction of the first dehydrated residue dehydroalanine (Dha) to 2-hydroxypropionate that causes an increase of 3 Da mass of homicorcin 1. Trypsin digestion of the core peptide and its variant followed by ESI–MS analysis suggests the presence of three ring structures, one in the N-terminal and other two interlocking rings at the C-terminal region that remain undigested. Homicorcin exerts bactericidal activity against susceptible cells by disrupting the integrity of the cytoplasmic membrane through pore formation as observed under FE-SEM.

## Introduction

Lantibiotics are ribosomally synthesized antimicrobial peptides possessing unusual amino acids normally not found in nature like lanthionine (Lan)- or methyllanthionine (MeLan)^1^. These peptides are mostly produced by a variety of Gram-positive bacteria and usually show antagonizing activity against Gram-positive bacteria, especially closely related species^2,3^. A few Gram-negative bacteria were also found to produce lantibiotics reported in recent years^4,5^. Producer strains synthesize lantibiotics as inactive prepeptides that consist of an N-terminal leader sequence and a C-terminal prepeptide part. This prepeptide sequentially undergoes several posttranslational modifications to become the mature lantibiotic. During the posttranslational modification unusual amino acids like Lan and MeLan are formed through intermolecular cyclization of the thiol groups of cysteine residues with Dha and Dhb, which are the dehydrated products of specific serine and threonine residues, respectively^2^. These thio-ether ring structures are assumed to provide the rigidity and impart resistance to proteolytic enzymes, temperature, pH and other parameters. Biosynthesis genes of lantibiotics are organized in the same gene clusters, which include the genes coding for the precursor peptides and other essential proteins involved in post-translational modification, processing of leader peptide, transportation, immunity, and regulation^6,7^. Recently, lantibiotics were classified in four classes based on the modification enzymes responsible for dehydration and cyclization^1^. In the class I lantibiotics, a dedicated dehydratase LanB, and a cyclase LanC perform dehydration and cyclization^1^. However, in class II lantibiotics, a biofunctional enzyme LanM introduces Lan or MeLan rings. For class III and class IV lantibiotics, tridomain proteins LanKC and LanL catalyze the formation of lanthipeptides, respectively^1^. Finally, cytoplasmic membrane protein LanT (an ATP-binding cassette [ABC] transporter) exports the modified precursor peptide outside the cell and an extracellular protease LanP cleaves the leader peptide to release the active lantibiotic. However, in some cases, a single protein LanT is responsible to export the precursor peptide and cleavage of leader peptide simultaneously^8^. Producer strains protect themselves from their own lantibiotic by expressing immunity proteins, including ABC transporter proteins LanFE(G) and/or lipoprotein LanI^9^.

Due to the increasing number of antibiotic resistance cases^10,11^, the world is in an urgent need for novel compounds and innovative methods to minimize the spread and development of drug resistant infection. Currently, among the *Staphylococcus aureus* strains isolated in the hospitals, 60–70% are found to be multidrug resistant^3,12^. Therefore, new antimicrobial drugs not affected by existing resistance mechanisms are needed to prevent the potential epidemic outbreaks of infectious diseases. The prototypic lantibiotic nisin has been utilized in the food industry as food preservative for over 40 years in more than 80 countries without the development of stable resistance, possibly as a consequence of its multiple modes of action^13^.

Potentially rich sources of lantibiotics are the endophytes, a treasure trove of therapeutically important bioactive compounds^14,15^. They are microorganisms, mostly fungi and bacteria that reside in plant tissue typically causing no apparent disease symptoms but on the contrary maintain a symbiotic relationship with host plants^16,17^. Endophytes basically have gained enormous attention for their capacity to control plant pathogenic insects and promote plant establishment and growth under adverse conditions^18–21^^.^ However, they are also reported to produce a plethora of bioactive secondary metabolites that have anti-arthritic, antimicrobial, anti-cancer, anti-diabetic, anti-insect, and immunosuppressant activities^22–25^. In particular, researchers have shown interest in antimicrobial peptides (AMPs) from different types of endophytes due to their chemical diversity and broad spectrum of activity^26,27^.

As lantibiotics have diverse applications, much attention has been paid in the last few decades on the identification of new peptides. In recent years, with the availability of abundant genomic sequence data in public databases, many new lantibiotics from different sources have been identified. In this study, we present a novel class I lantibiotic homicorcin and its variant homicorcin 1 isolated from a plant (jute) endophyte *Staphylococcus hominis* strain MBL_AB63. A large number of lantibiotics with diverse structure have been identified from different *Staphylococcal* strains. These include Pep5^28^ and epicidin 280^29^ from *Staphylococcus epidermidis* strains 5 and BN 280 respectively, epilancin K7^30^ and the closely related epilancin 15X^31^ from *S. epidermidis* strains K7 and 15X154, epidermin^32,33^ from *S. epidermidis* Tü3298, gallidermin^34^ from *Staphylococcus gallinarum* F16/P57 Tü3298, hominicin^35^ from *Staphylococcus hominis* MBBL 2–9, nukacin ISK-1^36^ and the very similar warnericin RB4^37^ from *S*. *warneri* ISK-1 and RB4 respectively, two component lantibiotic staphylococcin C55^38^ from *S. aureus* C55 and a few more. Most of the lantibiotics isolated from staphylococcal origin are found to be active against closely related species.The biosynthetic gene cluster of homicorcin possesses an additional oxido-reductase enzyme that further modifies the homicorcin N-terminal first residue of dehydroalanine to 2-hydroxypropionate forming homicorcin 1, a similar modification has been predicted for epicidin 280^29^ and epilancin 15X^31,39^. Homicorcin and its variant are equally produced in the culture supernatant without any induction. Further characterization of these two peptides reveals that they show almost similar spectrum of bactericidal activity against closely related species.

## Results

### In silico identification of the homicorcin gene cluster in *Staphylococcus hominis* strain MBL_AB63

**Figure 1:**
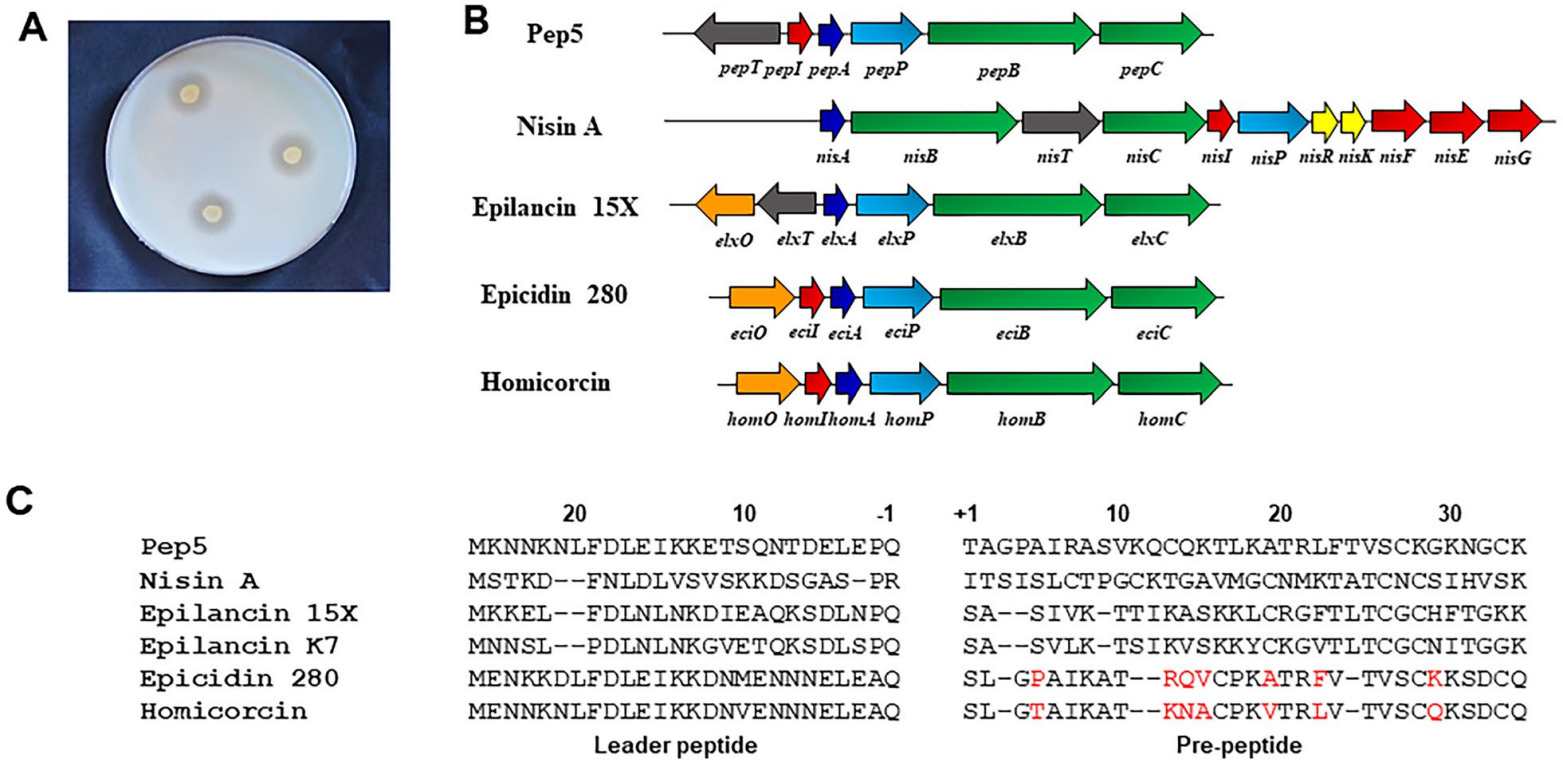
Prediction of the homicorcin gene cluster and comparison with other class I lantibiotics. (**A**) Inhibitory activity of *Staphylococcus hominis* strain MBL_AB63 (activity overlay assay against *Staphylococcus aureus* SG511). (**B**) Biosynthetic gene cluster of different class I lantibiotics. Homicorcin gene cluster was predicted using BAGEL 4.0 (*homO*: 3-oxoacyl-[acyl-carrier-protein] reductase gene; *homI*: immunity gene; *homA*: homicorcin gene; *homP*: protease gene for leader peptide cleavage; *homBC*: genes for post translational modification enzyme). (**C**) Comparisons between the peptide sequences of homicorcin and other class I lantibiotics (residues not similar to Epicidin 280 are shown in red).

Jute endophyte *Staphylococcus hominis* strain MBL_AB63 isolated in our laboratory showed significant antibacterial activity against *Staphylococcus aureus SG511* in a preliminary screening (Fig. 1A). Following whole genome sequencing of MBL_AB63 (DDBJ/ENA/GenBank accession number JAELVP000000000), the in silico tools BAGEL4 and anti-SMASH 5.0 were used to predict the biosynthetic gene cluster of the bioactive compound responsible for the antimicrobial activity. Both the tools identified a class I lantibiotic gene cluster of homicorcin that was found to be 82% similar to a reported lantibiotic; epicidin 280 with a difference of seven amino acids in the mature peptide (Fig. 1B,C).

The open reading frame (ORF) for the biosynthetic machineries for homicorcin was found to be organized in a single gene cluster where the structural gene (*homA*) is followed by the protease (*homP*), modification genes (*homBC*) and immunity gene *homI* in the same orientation (Fig. 1B). Homology of the homicorcin gene cluster products were compared using BLASTp and most of the gene products have high sequence similarity with epicidin 280 gene cluster products (Table 1). The *homA* gene appears to be the structural gene encoding a 56-amino acid precursor peptide that is cleaved between glutamine (Q) and serine (S) following post-translational modifications by *homBC*, to form a 30-residue mature homicorcin pre-peptide (Fig. 1C). A 3-oxoacyl-[acyl-carrier-protein] reductase gene (*homO*) is also present in the cluster that might be responsible for the reduction of the first modified serine residue (Dha) to 2-hydroxypropionate (Hpo) similar to the epicidin 280 gene cluster. The core peptide of homicorcin possesses three Ser, four Thr and three Cys residues. Among them all Ser residues and three Thr residues are predicted to be dehydrated by HomB. One N-terminal Thr, one C-terminal Thr and one C-terminal Ser dehydrated residues are predicted to be cyclized with the C-terminal Cys residues by HomC. Homicorcin leader peptide and pre-peptide were found to be structurally similar to nisin A, pep5, epilancin 15X and epicidin 280 (Fig. 1C).

**Table 1:** Open reading frame analysis of the homicorcin gene cluster using BLASTp.

Predicted ORFs	Amino acids	Protein homology (Gene bank accession number)	Identity in the aligned region	Expectation value
*homA*	56	EciA, epicidin 280 precursor peptide, *Staphylococcus epidermidis* (CAA74348.1) (56 aa)	46/56 (82%)	4e-25
*homB*	967	EciB, Lantibiotic dehydratase, *Staphylococcus epidermidis* (CAA74350.1) (976 aa)	803/967 (83%)	0.0
*homC*	353	EciC protein, Lanthionine synthetase C family protein, *Staphylococcus epidermidi*s (CAA74351.1) (397 aa)	331/353 (94%)	0.0
*homP*	300	EciP, Serine protease, *Staphylococcus epidermidis* (CAA74348.1) (300 aa)	243/300 (81%)	5e-176
*homO*	247	EciO, SDR family oxidoreductase, *Staphylococcus epidermidis* (CAA74346.1) (247 aa)	222/247 (90%)	1e-157
*homI*	62	EciI protein, Immunity protein, *Staphylococcus epidermidis* (CAA74347.1) (62 aa)	50/62 (81%)	2e-26

### Purification and MS/MS confirms the degree of dehydration of homicorcin

**Figure 2:**
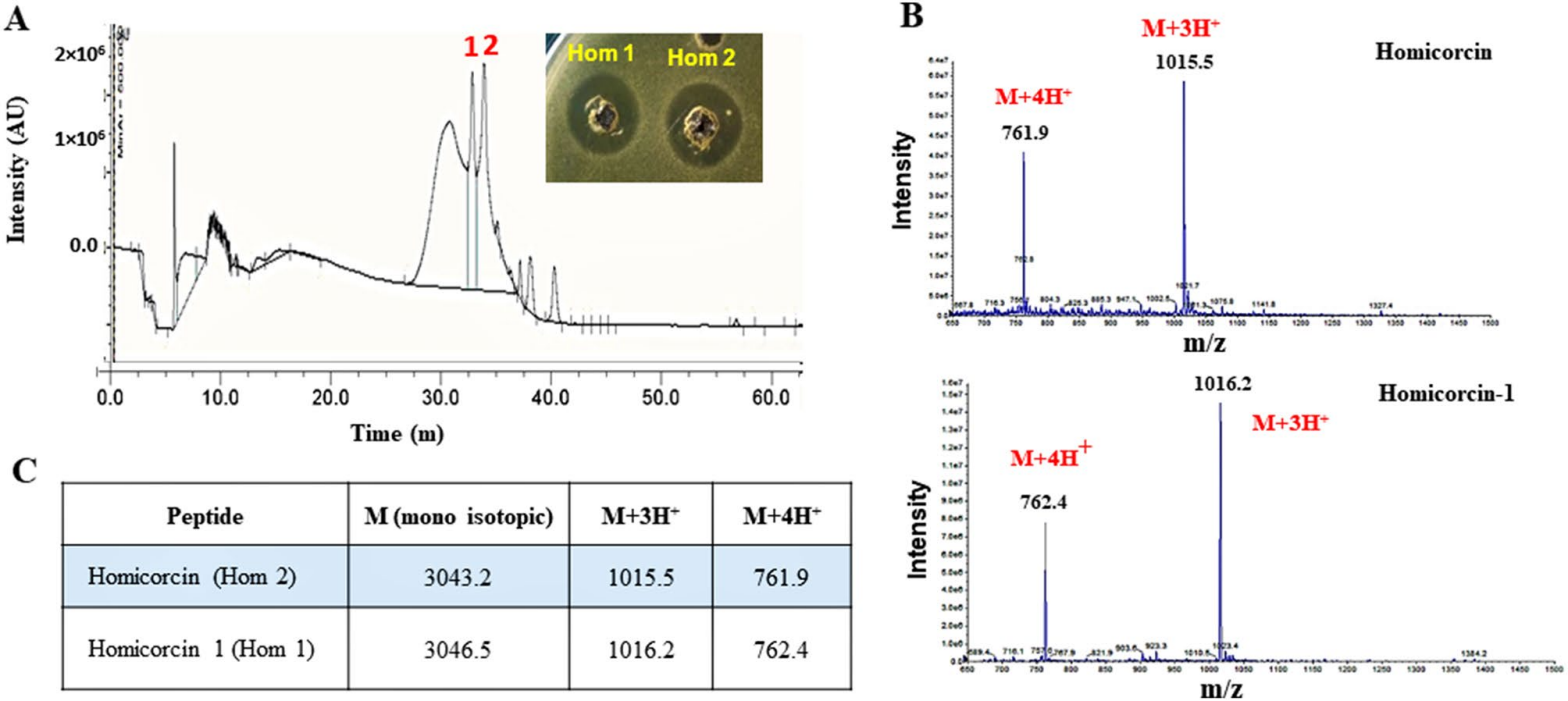
Purification of homicorcin through RP-HPLC and mass determination of active peaks by LC–MS. (**A**) RP-HPLC of active fractions (pooled from ion exchange); Two bioactive (peak-1 and peak-2) fractions were found eluting at 40% and 42% ACN gradient respectively . (**B**) Strong single peak found in each mass/charge (m/z) state. (**C**) Mass differences observed between homicorcin and its variant homicorcin 1.

An activity-guided purification was performed in four steps using ammonium sulfate precipitation extraction, size exclusion column chromatography, Sephadex cation exchange together with the C18 reversed phase-high performance liquid chromatography (RP-HPLC). Antimicrobial activity correlated with the peaks denoted as peak 1 and peak 2 eluted at 40% and 42% acetonitrile concentration respectively in the RP-HPLC chromatogram (Fig. 2A).

Completely purified antimicrobial fractions (peak-1, peak-2) produced by *S. hominis* strain MBL _AB63 was traced to the components with a strong signal at [M + 3H]^3^^3^^+^ = 1015.5 Da and 1016.2 Da respectively by liquid chromatography-mass spectrometry (LC–MS). LC–MS revealed that the corresponding active fractions have a mass of 3043.2 and 3046.5 Da respectively (Fig. 2B,C).

Correlating the predicted 3150 Da mass of homicorcin calculated from the genome sequence, gave insight into the degree of modification since the difference between these masses corresponded to the dehydration of six water molecules during maturation (18 Da reduction per dehydration). The active RP-HPLC fractions of peak 1 and peak 2 are named as homicorcin 1 and homicorcin respectively. Mass spectrometry revealed that homicorcin 1 is 3 Da larger than homicorcin in molecular mass (Fig. 2C).

### Trypsin digestion of purified peptides deduces the possible variations in post translational modification and thio-ether ring position

**Figure 3:**
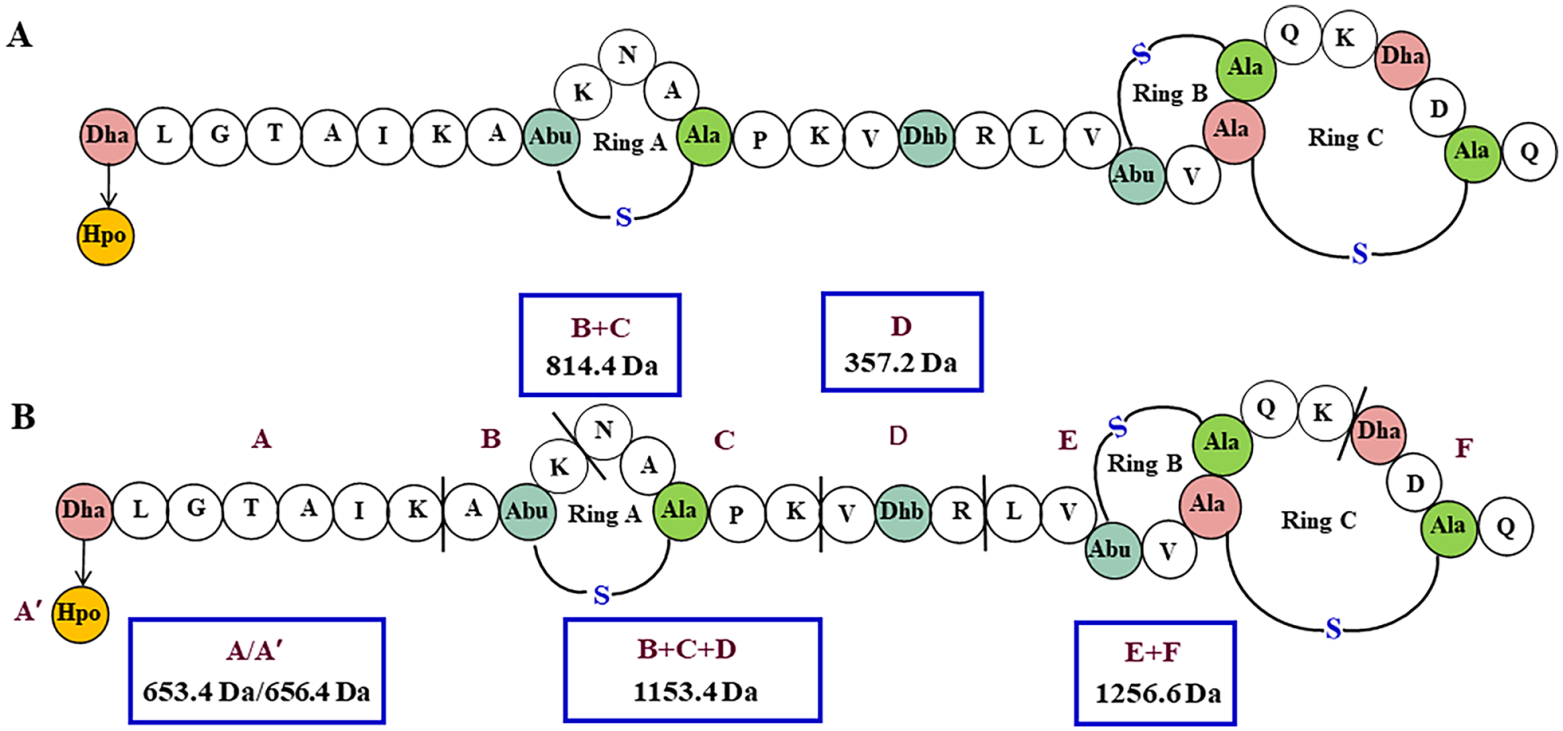
Proposed structure of Homicorcin. **(A)** Posttranslationally modified residues are indicated as follows: Dha,- dehydroalanine; Dhb—dehydrobutyrine; Abu—aminobutyric acid; Abu-S-Ala -Methyllanthionine; Dha-S-Ala – Lanthionine. The first residue Dha to be modified to 2-hydroxypropionate (Hpo) in homicorcin 1 is shown in a yellow circle. (**B**) Trypsin digestion sites of homicorcin and identified fragments by LC–MS (Trypsin digestion sites are indicated by (|) and digested fragments of the peptide are marked as **A**–**F**). The digested fragments obtained by trypsin digestion are (blue colored box) A- 653.4 Da, B + C- 814.4 Da, D- 357.2 Da, B + C + D- 1153.4 Da, E + F- 1256.6 Da. A’ is the modified N-terminal 2- hydroxypropionate (Hpo) fragment with a molecular mass of 656.4 Da (**B**).

The homicorcin leader peptide cleavage site and thio-ether ring patterns were predicted using RiPPMiner-Peptide webserver tool and found the cleavage site to be similar to class I lantibiotic cleavage site (Fig. 1C, Fig. S1) and the mature peptide contains three thio-ether rings as shown in Fig. 3A. Ring A is predicted to be between Thr9 and Cys13, ring B is between Thr21 and Cys24 and ring C is between Ser23 and Cys29 (Fig. 3A, Fig. S1).

In vitro fragmentation and sequencing of homicorcin and homicorcin 1 using Edman degradation poses a significant challenge as they contain several ring structures. The homicorcin possesses five trypsin digestion sites and hence de novo structure prediction using trypsin digestion was used to analyse the modification of amino acids and thio-ether ring positions (Fig. 1, Table 2). The digestion sites located between thio-ether bridges are usually not influenced by the peptidase, hence only five peptide fragments (fragment A, B + C, D, B + C + D, E + F) were found from the trypsin digestion product after RP-HPLC and LC–MS spectroscopy (Table 2, Fig. 3B, Fig. S2). The intensity of E + F fragment that contain predicted rings B and C was found to be very low and could not be reproduced. It should be noted that LC–MS abundances are peptide specific depending on the ionisation response. A potentially poor ionisation response of the fragments with ring structures could lead to underestimation of their abundance. Other tryptic peptide fragments could not be obtained through RP-HPLC possibly due to poor binding to the column or low abundance in the fragment mixture (Table 2). The fragment patterns predicted the position of three ring structures for these active peptides where ring one was found in fragment B + C and interlocking rings two and three were positioned in E + F peptide fragment.

**Table 2:** Possible tryptic peptides of homicorcin and its variant and their presence in MS analysis.

Peptide fragment	Domain	Tryptic peptide	m/z	Presence in MS
Homicorcin/ homicorcin 1				
A/A’	N-terminal	**S(de)**LGTAIK/**S(hpo)**LGTAIK	653.4/656.8	** + **
B	N-terminal	A**T(de)**K	301.2	**–**
C	N-terminal	NA**C**PK	532.2	**−**
B + C	N-terminal	A**T(de)**KNA**C**PK	814.4	** + **
D	Hinge	V**T(de)**R	357.2	** + **
B + C + D	N-terminal + Hinge	A**T(de)**KNA**C**PKV**T(de)**R	1153.5	** + **
E	C-terminal	LV**T(de)**V**S(de)C**QK	841.5	**−**
F	C-terminal	**S(de)**D**C**Q	434.1	**−**
D + E	Hinge + C-terminal	V**T(de)**RLV**T(de)**V**S(de)C**QK	1179.7	**−**
E + F	C-terminal	LV**T(de)**V**S(de)C**QK**S(de)**D**C**Q	1256.6	** + **
D + E + F	Hinge + C-terminal	V**T(de)**RLV**T(de)VS(de)C**QK**S(de)**D**C**Q	1594.8	**−**

A’ denotes fragment A of homicorcin 1 where dehydrated first Ser residue was modified to 2-hydroxypropionate (Hpo). Domain name represents the fragment position in the core peptide. Bold and underlined ‘S(de)’ or ‘T(de)’ residues are predicted to be dehydrated and ‘C’ are predicted to form thio-ether linkage with dehydrated S/T residues. . ( +) and (−) represents the presence or absence respectively of the fragment in MS analysis.

Besides the ring position, mass differences between the active peptides can also be explained from the trypsin digested fragments. The first dehydroalanine (Dha) in homicorcin is predicted to be oxidoreduced to 2-hydroxipropionate (Hpo) that causes a 3 Da mass increases in homicorcin 1. The *homO* gene product, an oxidoreductase similar to epicidin 280- associated *eciO* or epilancin 15X-associated *elxO* is responsible for the production of a mixture of peptides that have dehydroalanine (Dha) and 2-hydroxypropionyl (Hpo) groups at their N termini with a mass difference of 3 Da.

### Homicorcin shows inhibitory effects against various Gram-positive strains

Antimicrobial susceptibility testing of homicorcin was done against indicator strains consisting of *Staphylococcus simulans* 22*, Micrococcus luteus* ATCC1856*, Staphylococcus aureus* SG511*, Micrococcus luteus* DSM1790*, Lactococcus lactis* NCTC497*, Staphylococcus carnosus* TM300*,* methicillin resistant *Staphylococcus aureus* (MRSA), methicillin sensitive *Staphylococcus aureus* (MSSA1), *Bacillus subtilis* 168 and *Escherichia coli* DH5α (Table 3). Homicorcin was found to exhibit antimicrobial activity only against the Gram-positive bacteria, and not against Gram-negative ones.

**Table 3:** Antimicrobial susceptibility of homicorcin against different indicator strains.

Indicator strain	Inhibitory activity
*Staphylococcus simulans* 22	** + + + **
*Micrococcus luteus* ATCC1856	** + + **
*Staphylococcus aureus* SG511	** + + + **
*Micrococcus luteus* DSM1790	** + + **
*Lactococcus lactis* NCTC497	** + + **
*Staphylococcus carnosus* TM300	** + + **
Methicillin resistant *Staphylococcus aureus* (MRSA)	** + + **
Methicillin sensitive *Staphylococcus aureus* (MSSA1)	** + + **
*Bacillus subtilis* 168	** + **
*Escherichia coli* DH5α	**–**

Symbols represented as “ + + + ”, significant inhibition; “ + + ”, moderate inhibition; “ + ”, low inhibition and “–”, no inhibition.

The minimum inhibitory concentration (MIC) of homicorcin was compared with nisin A against *Staphylococcus simulans* 22, *Staphylococcus aureus* SG511*, Micrococcus luteus* ATCC1856, methicillin resistant *Staphylococcus aureus* (MRSA), methicillin sensitive *Staphylococcus aureus* (MSSA1) and *Escherichia coli* DH5α (Table 4). Homicorcin was found to be less potent than nisin A against the tested strains.

**Table 4:** Specific activities of homicorcin and nisin A.

	**MIC (μM)**	
**Indicator strain**	**Homicorcin**	**Nisin A**
*Staphylococcus simulans* 22	2.34	0.39
*Staphylococcus aureus* SG511	6.25	0.39
*Micrococcus luteus* ATCC1856	4.69	0.78
Methicillin resistant *Staphylococcus aureus* (MRSA)	36.0	12.5
Methicillin sensitive *Staphylococcus aureus* (MSSA1)	36.0	12.5
*Escherichia coli* DH5α	ND	ND

ND is for indicator growth inhibition not detected at the highest concentration of peptides used.

### Mode of antimicrobial mechanism of homicorcin and its variant

**Figure 4:**
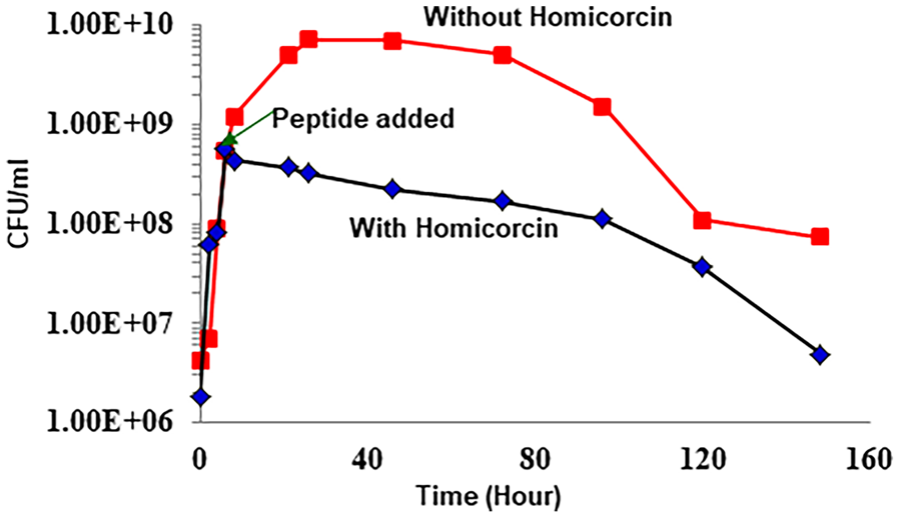
Antimicrobial mechanism of homicorcin against *Staphylococcus simulans* 22. Growth inhibition of *Staphylococcus simulans* 22 was measured as colony forming unit (CFU) count per ml for 148 h in both peptide treated and untreated samples. 8XMIC of homicorcin peptide was applied at mid-log phase.

To understand the antimicrobial mechanism of homicorcin, inhibitory effect was assessed against *Staphylococcus simulans* 22 in liquid culture for an extended period of 148 h. CFU/ml was calculated at regular time intervals for both the control and homicorcin treated sample. CFU count decreased significantly when homicorcin was added to the culture medium compared to the control one (Fig. 4). This result indicates that homicorcin has a bactericidal effect on target organisms.

**Figure 5:**
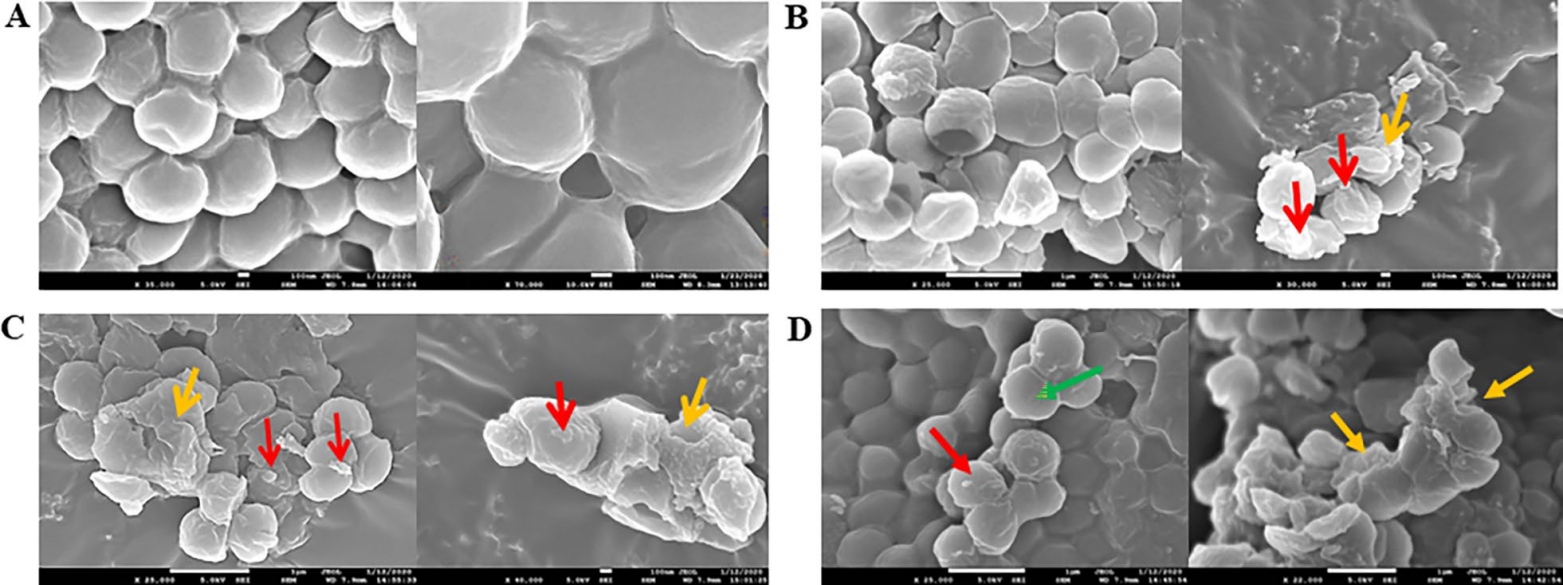
Electron microscopy of surface morphological changes of bacteria triggered by homicorcin and its variant homicorcin 1. Electron micrographs showing membrane morphology of *Micrococcus luteus* ATCC 1856 under the following conditions: (**A**) without treatment, (**B**) nisin A-treated and (**C**) homicorcin and (**D**) homicorcin 1 treated. {Normal cell (**green arrow**); Pore on cell membrane (**red arrow**); Lysed cell with deformed cell membrane (** orange arrow**)}.

To confirm the bactericidal effect of homicorcin and its variant against *Micrococcus luteus* ATCC1856, a field emission-scanning electron microcopy (FE-SEM) was performed. The indicator strain *Micrococcus luteus* ATCC1856 was treated with homicorcin, homicorcin 1 and nisin A under normal growth conditions. In all treated samples morphological changes were observed in the indicator strain with an induced membrane deformation that ultimately led to bacterial cell death. Peptide treated *Micrococcus luteus* ATCC1856 generated numerous spike-like membrane protrusions on the surface following drastically deformed cell membranes that resulted in the loss of intact cell shape and size (Fig. 5).

## Discussion

Endophytic bacteria are a source of a plethora of known and unknown novel biologically active metabolites^15^. The context of this study was set up with an aim to characterize a novel antimicrobial peptide (homicorcin) isolated from jute endophyte *Staphylococcus hominis* strain MBL_AB63. The preliminary assays for bioactive metabolites confirmed the production of a potential ribosomally synthesised antimicrobial peptide, commonly known as lantibiotic. Purification and characterization of this peptide was carried out using different quantitative and analytical methods including size exclusion, ion exchange and RP-HPLC chromatography and mass spectrometry. Scanning electron microscopy also helped us gain insights about the possible mode of action of homicorcin purified from *Staphylococcus hominis* strain MBL_AB63.

Lantibiotics are ribosomally synthesized antimicrobial peptides commonly produced by Gram-positive bacteria including genera such as *Bacillus, Enterococcus, Micrococcus, Streptococcus, Staphylococcus, Actinomycetes*, as well as some endophytic fungi^40^. However, lantibiotics produced by endophytic bacteria have not been reported so far. According to BACTIBASE (a database dedicated to bacteriocins), till now about 64 different lantibiotics have been reported; but only a few lantibiotics have been commercially applied or are under development for medical use in spite of their promising properties^41–43^. Research on lantibiotics has so far focused on bio-engineering to improve activity, to investigate the structure–activity relationship and to understand the modification process. Therefore, more focused research on clinical applications of lantibiotics is necessary to be carried out.

Lantibiotic biosynthesis requires the coordinated expression of a set of genes^44,45^. Whole genome sequence analysis for secondary metabolites of *S. hominis* strain MBL_AB63 identified a complete class I lantibiotic gene cluster. The gene cluster contains five genes (*homABCOP*) involved in biosynthesis of homicorcin, and one gene potentially involved in immunity (*homI*) (Table 1; Fig. 1B). The cluster organization resembles that of the lantibiotics, epicidin 280^29^, epilancin 15X^39^, and pep5^46^ produced by different strains of staphylococci*,* suggesting that these clusters have evolved from a common ancestor. Our predicted peptide HomA has high amino acid sequence similarity (82%) to the epicidin 280 precursor peptide (EpiA) with seven amino acid differences (Table 1)^29^. HomA contains 26 amino acids in its N-terminal region as leader peptide and 30 amino acids in its C-terminal pre-peptide region. The leader region also contains the conserved motif F-(N/D)-L-(N/D/E) and an Ala at position -2 that are characteristic of class I lantibiotics^47^ (Fig. 1C). Downstream of HomA, there is a putative serine protease HomP which has high amino acid sequence similarity to EciP, the protease involved in the biosynthesis of epicidin 280 (Table 1)^29^. HomP likely removes the leader peptide before the mature peptide is transported outside the cell, similar to NisP or EpiP that are extracellularly located and remove the leader region once the peptide has been secreted. The cluster also contains HomB, downstream of HomP, which most likely catalyzes the dehydration of Ser or Thr residues of the HomA C-terminal pre-peptide region. Three Ser and three Thr residues are predicted to be dehydrated by HomB. The ORF for *homC* encodes a protein with high amino acid sequence similarity to EciC, the cyclase responsible for Lan and MeLan ring formation in epicidin 280 (Table 1). Comparing the sequence similarity of homicorcin and closely related peptides it can be predicted that homicorcin contains one Lan and 2 MeLan ring structures in its mature form. Based on the lantibiotic maturation pathway, for homicorcin we can predict that the precursor peptide HomA is modified by the dehydratase HomB and the cyclase HomC to produce the cross-linked peptide. Then, the leader peptide is removed by the protease HomP, producing an N-terminal dehydroalanine (Dha) present in equilibrium with a reduced form of Dha to 2-hydroxypropionate (Hpo) by the enzyme HomO^29,39^. The HomO of homicorcin similar to the oxidoreductase EciO (Table 1) is hypothesized to be involved in the reduction of N-terminal free Dha to 2-hydroxypropionate (Hpo) in the biosynthesis of homicorcin 1^29^. The role of the N-terminal Hpo in homicorcin 1 is currently unknown. However, N-terminal modifications are common in lantibiotics and include (methyl)lanthionines, disulfides, pyruvate and lactate groups, 2-oxobutyrate groups, and acyl groups. These modifications might protect the terminal residues from exoproteases^48,49^. The homicorcin gene cluster also possesses a putative immunity gene, *homI* to protect the producer strain from its own antibiotic. HomI shows 81% amino acid similarity with EciI, the protein responsible for providing immunity to *Staphylococcus epidermidis* against epicidin 280 (Table 1)^29^. The Pep5 producing strain also gets protected by the PepI immunity protein which has 72% sequence similarity with EciI^29^. Therefore, the producer self-protection mechanism is likely to be mediated by HomI and could be based on the same molecular mechanism as the producer self-protection mediated by EciI and PepI.

Purified homicorcin demonstrated stability in a broad range of pH values (pH 4.0 – 10.6) and retained activity even after treatment at 100 °C (data not shown). Although they showed stable activity under biological pH but the activity began to decrease slightly around pH 10.6. Its stability in a broad range of pH values corroborates with previous studies which show that lanthionine rings and dehydrated residues included in a lantibiotic’s structure are highly stable at low pH^50^. Homicorcin was found to exhibit antimicrobial activity against closely related Gram-positive bacteria. Among the indicator strains tested homicorcin showed potent activity against *Staphylococcus* spp. and *Micrococcus* spp. including MRSA (methicillin-resistant *Staphylococcus aureus*) and MSSA1 (methicillin-susceptible *Staphylococcus aureus*) strains. Lantibiotics isolated from *Staphylococcal* spp. are mostly active against different strains of the same group^3^. Among 16 test strains of *S. aureus*, Pep5 and epidermin were found to inhibit 14 and 13 strains respectively including Brazilian MRSA clone A/22C^51^. Gallidermin have bactericidal activity against both MRSA and MSSA strains^52^. Hominicin displayed potent activity against MRSA ATCC 11,435, and vancomycin-intermediate *S. aureus* CCARM3501^35^. Nukacin ISK-1 is active against a wide range of Gram positive bacteria including *Bacillus* and *Lactobacillus* strains^53^.

The position of the thio-ether rings in the mature homicorcin peptide was predicted using RiPPMiner-Peptide webserver tool which identified three rings, one before the hinge and other two are interlocking at C-terminal region, quite similar ring patterns to those predicted for epicidin 280 as well^29^. Trypsin digestion of the core peptide followed by ESI–MS analysis of the digested fragments has been proven to be a useful tool for analysing modifications and ring topology of different lantibiotics in vitro ^54–58^. Trypsin is the most common protease for generating peptides for MS analysis. Information regarding ring topology is based on the suppression of fragmentation within the rings. Both homicorcin and homicorcin 1 contain five trypsin cleavage sites, two of them are directly adjacent to potential ring structures (Table 1, Fig. 3B). It has been reported that in this case the sites are protected against trypsin cleavage^59^. Digested fragments of homicorcin and its variant indicate that trypsin cleavage sites are efficiently cleaved except for the sites within the ring structures (Fig. 3B, Table 1). The first tryptic peptide at the N-terminus of homicorcin and its variant was found to have a 3 Da mass difference that strongly correlates with the modification of initial Dha to 2-hydroxypropionate (Hpo) of homicorcin 1.

To understand the mode of action of homicorcin, a growth inhibition assay was performed against *Staphylococcus simulans* 22. *Staphylococcus* cell viability counts clearly indicated that peptide treated indicator strain showed significant decrease of viable cells as measured by CFU count over time. Both nisin and pep5 which belong to class I lantibiotic group also show similar bactericidal effect against the target organisms^13,60^. Cellular morphology of homicorcin treated cells assessed under field emission-scanning electron microscope (FE-SEM) clearly indicated damage on the cell surfaces of treated indicator strain and also shown rapid leakage of K^+^ ion in the surroundings (data not shown). Homicorcin is a cationic peptide having five positively charged amino acids. Therefore, it can be predicted that like other small cationic peptides, homicorcin can target the cytoplasmic membrane of sensitive cells^61–63^, where they act to dissipate the proton motive force (PMF) through the formation of discrete pores in the cytoplasmic membrane, and thus deprive cells of an essential energy source^64^. This effect most likely leads to an efflux of small molecules (potassium and amino acids) and results in the arrest of all cellular biosynthesis. Conformational studies of different lantibiotics have shown that, although the peptides are flexible in an aqueous environment^65,66^, in the presence of a lipophilic environment they adopt an amphipathic conformation with a central hinge region^67^, which enables the peptides to insert into the bacterial membrane, introduce temporary membrane perturbations and assemble into a pore. Lantibiotics also target the cell wall component lipid II as an alternative mode of action and inhibit cell wall biosynthesis^68,69^. Although little is known of lantibiotic resistance in comparison to resistance to commercial antibiotics, a few reports have shown that modification of cell wall composition of teichoic acids due to *dltA* gene alteration^70^, change of expression of penicillin binding proteins (PBPs)^71^, composition of cell membrane^72^ and presence of two-component sensing systems in target organisms like BceRS in *B. subtilis*^73^, BraRS of *S. aureus*^74^*,* AnrAB transporter of *L. monocytogenes*^75^, VraSR of *S. aureus*^76^ may greatly influence the development of lantibiotic resistance.

In conclusion, the newly identified class I lantibiotic homicorcin produced by *Staphylococcus hominis* strain MBL_AB63 has potent antimicrobial activity against Staphylococci and related species. Moreover, biosynthetic mechanism of this peptide is interesting compared to other reported lantibiotics. Heterologous expression of the homicorcin gene cluster, the current focus of our lab, will provide an in-depth understanding of the role of individual genes involved in the biosynthesis together with a comprehension of the structure function relationship of this peptide. The N-terminal modification of homicorcin might provide the peptide with higher stability and activity.

## Material and methods

### Homicorcin producer strains and in silico prediction of the lantibiotic gene cluster

The homicorcin producer strain *Staphylococcus hominis* strain MBL_AB63 was isolated as a jute endophyte in the Molecular Biology Laboratory (MBL), Dept. of Biochemistry and Molecular Biology, University of Dhaka. Whole genome sequencing of MBL_AB63 has been carried out using Illumina MiSeq platform and the genome sequence submitted to NCBI (DDBJ/ENA/GenBank accession number JAELVP000000000) database. For in silico prediction of lantibiotic biosynthesis, BAGEL4 and anti-SMASH 5.0 automated tools were used.

### MBL_AB63 culture and homicorcin crude preparation

A single colony of *Staphylococcus hominis* strain MBL_AB63 was cultured overnight in 5 ml TSB (Tryptic Soy Broth) (OXOID, England) liquid broth at 37 °C. 4.0 ml of overnight culture was added in 1.0 L freshly prepared TSB broth to obtain an OD_600_ at 0.01 for large scale peptide production and incubated in a shaking incubator for 24 h at 37 °C. As the desired antimicrobial peptide is part of the extracellular proteins, cell free supernatant was collected after centrifugation at 7000 rpm for 10 min at 4 °C. The antimicrobial peptide was extracted and precipitated using 60% ammonium sulfate at 4 °C. The mixture was then centrifuged at 8000 rpm for 30 min at 4 °C and the pellets were dissolved in sterile nano-pure water. Antimicrobial activity was assayed using *Staphylococcus simulans* 22 as an indicator strain.

### Purification of homicorcin and its variant using column chromatography

An activity guided purification of homicorcin and its variant were performed simultaneously using a discrete column chromatography method. Ammonium sulfate precipitated crude protein was applied to a Sephadex G-50 fine (Pharmacia, Uppsala, Sweden) size exclusion column previously equilibrated with 10 mM sodium phosphate buffer containing 50 mM NaCl of pH 6.0 and eluted with the same buffer at a flow rate of 1.0 ml/min. Gel filtrated active pooled fractions were diluted three times with 10 mM sodium phosphate buffer (pH 6.0) and passed through a weak cation exchanger CM Sephadex C-25 (Pharmacia, Uppsala, Sweden) column at a 1.0 ml/min flow rate. The column was washed with 10 mM sodium phosphate buffer (pH 6.0), and active peptides bound to the resin were eluted with 200 ml 10 mM sodium phosphate buffer with 1 M NaCl solution in a linear gradient manner. Reverse phase-high performance liquid chromatography (RP-HPLC) was used as the final purification step. Active pooled samples obtained from ion exchange were injected into RP-HPLC to get completely purified peptide using a multistep gradient using acetonitrile and nano-pure water with 0.05% TFA as mobile phase. Before RP-HPLC separation, all the organic solvents were filtered through polytetrafluoroethylene (PTFE) organic filter (Ultipor® N®66 Nylon 6, 6membrane, 0.45 μm) and deaerated. A reverse phase C18 column (Luna 5u C18 100A, 250 × 10.0 mm particle size 5 μm, pore size 110 A) was used and the peaks were recorded by UV (DAD – Diode Array Detector) detection at 220 nm.

### Prediction of homicorcin leader peptide cleavage site and thio-ether cross links

“RiPPMiner-Peptide” tool was used to identify the homicorcin leader peptide cleavage site, cross links and similarity with different RiPPs (ribosomally synthesized and post-translationally modified peptides). RiPPMiner is a machine learning based webserver for deciphering chemical structures of RiPPs. It derives its predictive power using a manually curated database of more than 500 + experimentally characterized RiPPs belonging to 13 subclasses. These classes include lantipeptide, bottromycin, cyanobactin, glycocin, lasso peptide, linearazol, microcin, sactipeptide, thiopeptide, auto inducing peptide etc.

### Mass spectrometry analysis (LC–MS/MS)

The LC–MS measurement was performed on a platform of Agilent 6420 LC/TQ, equipped with 1290 Infinity II LC System utilizing an Agilent rapid resolution HD ZORBAX Eclipse Plus C18 column (2.1 X 50 mm, 1.8-μm particle size) and sample purity was monitored by the 1290 Infinity II Diode Array Detector FS at 220 nm and 254 nm. MS measurement was carried out using the standard ESI (electrospray ionization) source that was equipped with turbo ion spray source operating at 350 °C with the fragmentation voltage set to 135 V and collision energy at 35 V.

Mass-spectra were acquired in centroid mode ranging from 100–2000 m/z in positive ionization mode with an auto MS2 fragmentation. Optimization of the MS parameters was conducted using standard solutions of each analyte prepared in acetonitrile: water (1:1) with 0.1% HCOOH and infused at a flow rate of 0.2 ml/min.

### De novo peptide sequencing using trypsin digestion

Trypsin is a serine peptidase that predominantly cleaves proteins at the carboxyl side of the amino acids lysine (K) and arginine (R) except when either is bound to a C-terminal proline residue. Trypsin (Gibco ™ Trypsin dissolved in 0.2 N HCl) was added to the peptide solution as a final peptide: protein ratio of 1:20 (w/w) where it was desirable that protein concentration is at least 0.1 mg/ml. Then the sample was incubated overnight at 37 °C. Incubated samples were then applied in RP-HPLC and LC–MS to identify the digested fragments.

### Homicorcin susceptibility test

On tryptic soy agar (TSA) plates, indicator strains with appropriate concentrations (10^6^ cells/ml) were overlaid using TSB soft agar (3% TSB and 0.8% agar). Indicator strains used for susceptibility test were *Staphylococcus simulans* 22*, Micrococcus luteus* ATCC1856*, Staphylococcus aureus* SG511*, Micrococcus luteus* DSM1790*, Lactococcus lactis* NCTC497*, Staphylococcus carnosus* TM300*, Escherichia coli* DH5α. Wells were made over the soft agar and 30 μl of purified peptide was applied. After overnight incubation, wells containing the peptides with antimicrobial activities inhibited the growth of the test strains by forming clear zones around the wells.

### Determination of minimum inhibitory concentration (MIC)

The MIC of homicorcin and nisin A were determined twice for each strain by spotting on a lawn antimicrobial assay. The method which has been described for the disk diffusion assays in the National Committee for Clinical Laboratory Standards protocol was used for the preparation of the plate^77^. The inoculum was prepared by suspension of colonies in sterile solution of TSB, grown overnight and the optical density was adjusted to 0.5 McFarland standard (1 × 10^8^ cells/ml). Homicorcin and nisin A were diluted to obtain concentrations in the range 100.0–0.19 µM, and the MIC was determined after 16 h of incubation at 37 °C.

### Determination of bactericidal/bacteriostatic activity

To determine the bacteriostatic or bactericidal nature of homicorcin, *Staphylococcus simulans* 22 was used as an indicator strain. 8XMIC of homicorcin was applied at the mid-log phase of indicator growth. The colony-forming unit (cfu/ml) was determined for 148 h in both peptide treated and untreated samples. CFU was counted by the spread plate method after plating the cells on TSA plate in tenfold serial dilutions.

### Electron microscopy of homicorcin treated bacterial cells

For insights into the surface morphology of *Micrococcus luteus* ATCC1856 upon treatment with homicorcin and its variant, the bacterial samples were visualized under field emission-scanning electron microscope (FE-SEM) (model Jeol-JSM 7610F). For sample preparation, bacteria were collected in early stationary phase, incubated for growth upon treatment with 5 × MIC of homicorcin and its variant. 1 mL of each bacterial sample was centrifuged at 5000 rpm for 5 min at 4 °C and the pellet obtained was washed twice with 1X PBS buffer (pH-7.4). After washing, pellets were fixed by incubating cells in 0.25% glutaraldehyde in Na-phosphate buffer for 30 min. The fixed pellet was washed with 10 mM Na-phosphate buffer. Sequential dehydration was done using 30%, 50%, 70%, 90%, and absolute ethanol. These dehydrated samples were coated by platinum and visualized under FE-SEM.

## Supplementary Information

Supplementary Information.
