# Supplementary material for: A plant endophyte Staphylococcus hominis strain MBL_AB63 produces a novel lantibiotic, homicorcin and a position one variant
Source: Sci Rep. 2021 May 27;11:11211. doi: 10.1038/s41598-021-90613-9 (PMC8159966; doi:10.1038/s41598-021-90613-9)
Supplement: Supplementary file 1 — Supplementary Information. [file 41598_2021_90613_MOESM1_ESM.pdf]

A plant endophyte *Staphylococcus hominis* strain MBL_AB63 produces a novel lantibiotic, homicorcin and a position one variant

M. Aftab Uddin[1, 2,¶], Shammi Akter[1,¶], Mahbuba Ferdous[1, 3], Badrul Haidar[1, 4], Al Amin[1], A. H. M. Shofiul Islam Molla[5], Haseena Khan[1,*], Mohammad Riazul Islam[1,*]

A. Homicorcin class, cleavage site and cross links prediction

Predicted Class: LanthipeptideA

Cleavage site (12mer): N E L E A Q ▬ S L G T A I

Leader peptide: M E N N K N L F D L E I K K D N V E N N N E L E A Q

Core peptide: S L G T A I K A T K N A C P K V T R L V T V S C Q K S D C Q

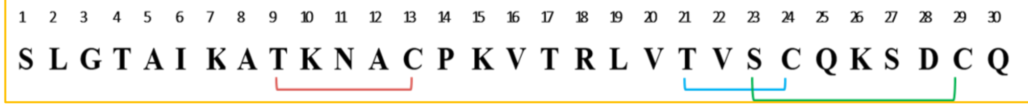

B. Homicorcin prepeptide sequence similarity with different RiPPs

Subject	Identity (%)	Alignment length	e-value
Epicidin 280	82.1	56	9.00E-31
Pep5	57.4	54	3.00E-16
Epilancin 15X	37.0	46	2.00E-04
Paenicidin A	34.3	35	0.21
Paenicidin B	30.4	23	0.44
Streptin 2	25.5	47	0.58
Gallidermin	25.0	44	0.80
Elgicin	27.3	55	2.70
Epidermin	22.2	45	3.60
Nukacin ISK 1	50.0	12	4.30
Nukacin A	50.0	12	4.30
Prochlorosin 4.3	39.1	23	6.50
FlvA2.d	28.6	14	6.60
Salivaricin A2	26.8	41	6.80
Sap B	83.3	6	6.80

Figure S1: Prediction of homicorcin class, cleavage site, cross links and similarity with different RiPPs (ribosomally synthesized and post-translationally modified peptides) using RiPPMiner-Peptide webserver tool. (A) Homicorcin possesses class I lantibiotic cleavage site and three thio-ether rings. (B) Epicidin 280 has the highest sequence similarity with homicorcin prepeptide (HomA).

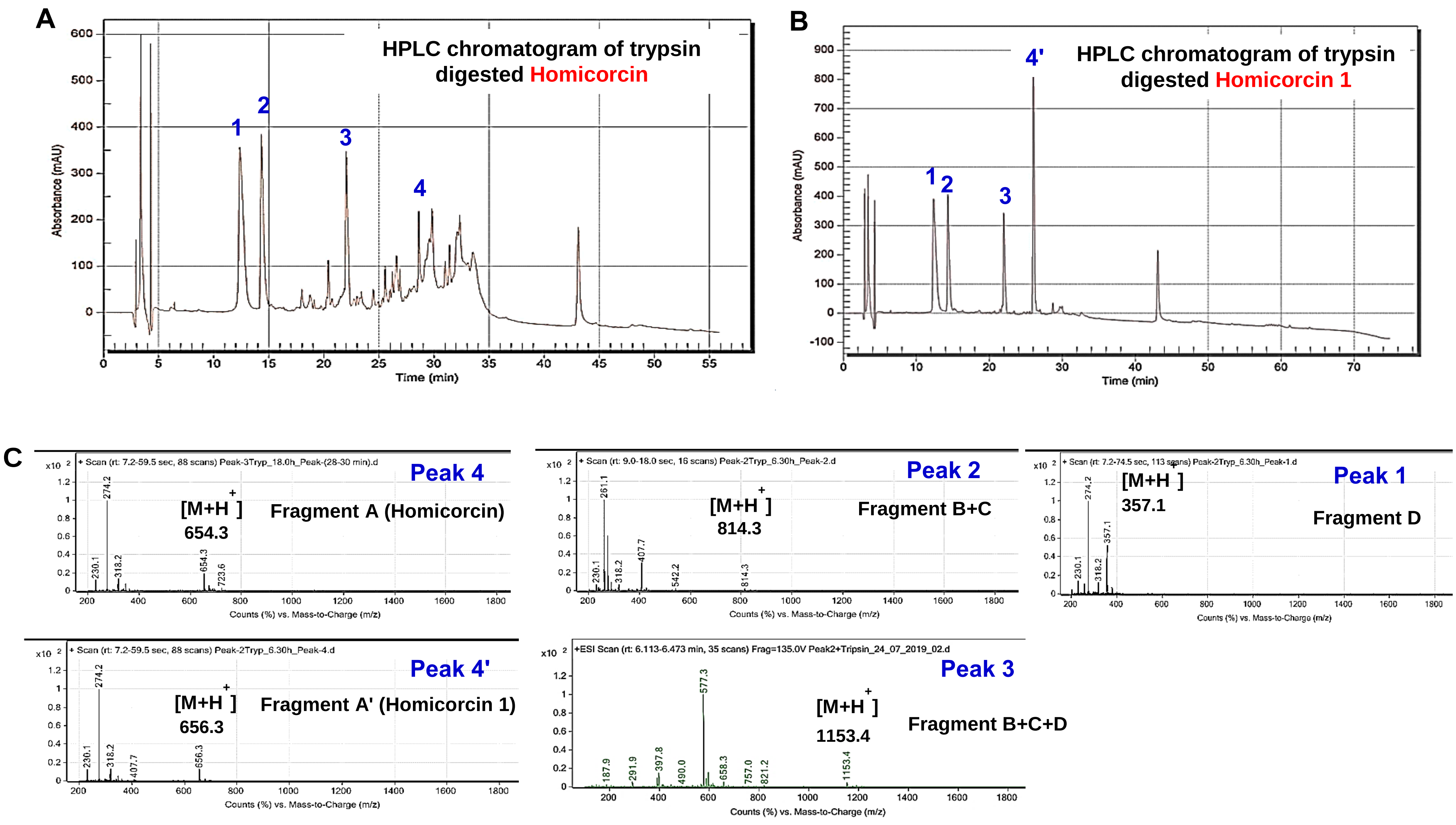

Figure S2: Trypsin digestion of homicorcin and homicorcin 1. (A & B) HPLC separation of trypsin digested fragments of homicorcin and homicorcin 1. (C) ESI-MS of selected peaks from HPLC representing different mass fragments of homicorcin and homicorcin 1 as shown in figure 3B**.**
